# Crystal structure of human CK2α at 1.06 Å resolution

**DOI:** 10.1107/S0909049513020785

**Published:** 2013-10-02

**Authors:** Takayoshi Kinoshita, Tetsuko Nakaniwa, Yusuke Sekiguchi, Yuri Sogabe, Atsushi Sakurai, Shinya Nakamura, Isao Nakanishi

**Affiliations:** a Graduate School of Science, Osaka Prefecture University, 1-1 Gakuen-cho, Naka-ku, Sakai, Osaka 599-8531, Japan; b Institute for Protein Research, Osaka University, 3-2 Yamadaoka, Suita, Osaka 565-0871, Japan; c Department of Pharmaceutical Sciences, Kinki Univeristy, 3-4-1 Kowakae, Higashi-Osaka, Osaka 577-8502, Japan

**Keywords:** CK2 kinase, catalytic subunit, high-resolution crystal structure

## Abstract

The Ser/Thr kinase CK2 consists of two catalytic subunits (CK2α) and a dimer of the regulatory subunits (CK2β), and is a ubiquitous enzyme that regulates growth, proliferation and the survival of cells. CK2 is a remarkable drug target for potentially treating a wide variety of tumours and glomerulonephritis. The purified CK2α protein was crystallized using ethylene glycol as a precipitant. The crystal structure of CK2α with 21 loci of alternative conformations, including a niacin, 19 ethylene glycols and 346 waters, was determined at 1.06 Å resolution to an *R*_work_ of 14.0% (*R*_free_ = 16.5%). The alternative ensemble in the internal hydrophobic core underpins the plasticity of the αD-helix responsible for the regulation of ATP/GTP binding. The clear density map indicates that a niacin molecule, contained in the *Escherichia coli* culture medium, binds to the ATP binding site. An ethylene glycol molecule binds in the hydrophobic pocket lateral to the αD-helix forming the rim of the active site. The other ethylene glycol molecules occupy physiologically significant sites, including the CK2β binding interface and substrate binding site, as well as the gap in the crystal packing. Together with water molecules in the active site, these structural insights should facilitate drug discovery.

## Abstract 2

The high-resolution crystal structure of the human CK2 catalytic subunit reveals the structural insights that probably promote drug discovery.

## 1. Introduction

CK2 (previously called casein kinase 2) is a ubiquitously expressed serine/threonine kinase that facilitates the growth, proliferation and survival of a variety of cells. The CK2 holoenzyme consists of two catalytic subunits (CK2α) and two regulatory subunits (CK2β) (Niefind *et al.*, 2001 ▶). CK2α has no phosphorylation site required for activation and thus is constitutively active with or without CK2β (Mazzorana *et al.*, 2008 ▶). CK2 is pleiotropic with more than 1000 *in vivo* substrates with preference towards the acidic residues at the P+1 and P+3 positions, *i.e.* the adjacent and third residues of the phosphorylation residue (P+0 position) (Meggio & Pinna, 2003 ▶). The kinase uses either GTP or ATP as a phosphor donor in the kinase reaction (Niefind *et al.*, 1999 ▶). This unusual dual specific potency was illustrated by the characteristic large space corresponding to the ribose binding site around the stretch from Asn118 to Asp120, and the hydrophobic residues Met163 and Val66, as well as the flexibility of the αD-helix (Niefind *et al.*, 1999 ▶).

CK2 represents an important target protein for tumour therapy owing to overexpression in various tumours (Duncan & Litchfield, 2008 ▶). In addition, it is a target protein for glomerulonephritis therapy, which is supported by experiments showing that administration of an antisense oligodeoxynucleotide against CK2 or low-molecular-weight CK2-specific inhibitors effectively prevent the progression of the renal disease in a rat model (Yamada *et al.*, 2005 ▶). Several planar chemical or natural compounds have been identified as ATP-competitive selective CK2 inhibitors, including ellagic acid (Cozza *et al.*, 2006 ▶), emodin (Raaf *et al.*, 2008*a* ▶), apigenin (Critchfield *et al.*, 1997 ▶), 4,5,6,7-tetrabromo-1*H*-benztriazole (Sarno *et al.*, 2001 ▶), 5-oxo-5,6-dihydroindolo[1,2-*a*]quinazolin-7-yl (Vangrevelinghe *et al.*, 2003 ▶) and 5,6-dichloro-1-β-d-ribofuranosylbenzimidazol (DRB) (Zandomeni *et al.*, 1986 ▶). Other trials to produce potent and selective CK2 inhibitors are currently in progress (Suzuki *et al.*, 2008 ▶; Nie *et al.*, 2008 ▶; Hou *et al.*, 2012 ▶). In the process of a lead optimization stage, expectant lead structures are modified by systemically changing functional groups or side chains on a given core scaffold. The crystal structures of human CK2α complexed with ATP-competitive inhibitors, including emodin (Raaf *et al.*, 2008*a* ▶), DRB (Raaf *et al.*, 2008*b* ▶), ATP analogues (Ermakova *et al.*, 2003 ▶; Niefind *et al.*, 2007 ▶; Ferguson *et al.*, 2011 ▶) and ellagic acid (Sekiguchi *et al.*, 2009 ▶) provide valuable starting points that guide the selection of an appropriate functional group to be attached or modified. In order to establish the correct ration­ale for structure-based drug design on CK2, we have evaluated the binding features of homologous compounds by calorimetric medium-resolution structural and computational analyses (Kinoshita *et al.*, 2011 ▶).

Here, we determined the highest-resolution crystal structure of CK2α at 1.06 Å resolution, which reinforced a functional mechanism for the αD-helix and should facilitate drug discovery.

## 2. Methods

### 2.1. Preparation of the human CK2 catalytic subunit (CK2α)

Recombinant human CK2α was prepared basically according to the reported protocol (Kinoshita *et al.*, 2011 ▶). The C-terminal truncated form of CK2α was cloned into the pGEX6P-1 expression vector (GE Healthcare) and expressed in the transformed *Escherichia coli* strain HMS174 (DE3) cells as a GST-fused protein at the N-terminus. Cells were cultured in Luria-Bertani medium supplemented with ampicillin at 310 K. Expression was induced by the addition of isopropyl-1-thio-β-d-galactopyranoside to a final concentration of 0.2 m*M* when cells had reached an optical density 600 nm reading of 0.8. The cells were grown for a further 15 h at 298 K. The cells were harvested, resuspended in a buffer consisting of 150 m*M* NaCl and 25 m*M* Tris-HCl, pH 7.4, and sonicated. After removing the cellular debris by centrifugation, the supernatant was loaded onto glutathione Sepharose 4B resin (GE Healthcare) and incubated at 277 K for 1 h. The slurry was washed with cleavage buffer containing 50 m*M* Tris-HCl, pH 8.0, 150 m*M* NaCl, 1 m*M* EDTA, 5 m*M* dithiothreitol and 0.1% Tween-20. The GST-fused CK2α protein was digested with 80 U ml^−1^ PreScission protease (GE Healthcare) for 4 h at 277 K. The disengaged GST-free CK2α protein was rebuffered with the MonoQ buffer consisting of 25 m*M* Tris-HCl, pH 8.0 and 5 m*M* dithiothreitol. CK2α was further purified by anion-exchange chromatography with a MonoQ column on an AKTA Explorer system (GE Healthcare) using a linear NaCl gradient of 0 to 0.5 *M* of the MonoQ buffer during 25 column volumes at 277 K.

### 2.2. Crystallization and structural analysis of CK2α

The purified protein was concentrated to 5 mg ml^−1^ in the MonoQ elution buffer and used for crystallization. The prism-shaped crystals of CK2α were obtained by the sitting-drop vapour-diffusion method using 20–25% ethylene glycol as the precipitant. This condition used was the same as the previous result (Sekiguchi *et al.*, 2009 ▶). A single crystal was set onto the goniometer head with a distance of 95.8 mm to the Quantum 270 CCD-detector (ADSC, Poway, CA, USA) and directly flash-frozen using a nitrogen gas stream cooled at 95 K in the BL17A station of the Photon Factory, Tsukuba, Japan. The monochromated X-ray radiation with a wavelength of 0.98 Å was exposed to the crystal in 6 s oscillating with a 0.5° width. The diffraction derived from this condition was collected on an image. A total of 360 images were collected and processed with the program *HKL2000* (Otwinoski & Minor, 1997 ▶). The structure of CK2α was solved by the molecular replacement method using the 1.6 Å resolution apo structure (Kinoshita *et al.*, 2011 ▶) as a starting model with the program *MolRep* (Vagin & Teplyakov, 2000 ▶) in the CCP4 suite (Collaborative Computational Project, Number 4, 1994 ▶). All refinements and model modifications were performed using the programs *Coot* (Emsley & Cowtan, 2004 ▶) and *Refmac5* (Winn *et al.*, 2003 ▶). Data collection and refinement statistics are shown in Table 1 ▶. The final set of coordinates was deposited into the Protein Data Bank with the accession code 3war.

### 2.3. Binding energy calculation

As a candidate ligand which is assignable to the electron density at the ATP binding site, three molecules, nicotinic acid, nicotinamide and benzamidine, could be considered. To deduce the most plausible ligand, binding energies of these molecules were calculated by the molecular mechanics Poisson–Boltzmann surface area (MM-PBSA) method (Kollman *et al.*, 2000 ▶). Each complex structure was modelled using *MOE* (Chemical Computing Group, Montreal, Canada) based on the atomic coordinates of 3war. Two symmetric orientations, both of the pyridine ring of niacin and of the carboxamide group of nicotinamide, were considered. Therefore, two and four distinct orientation models were built for nicotinic acid and nicotinamide, respectively. The GAFF (Wang *et al.*, 2004 ▶) and Amber ff99SB (Hornak *et al.*, 2006 ▶) force fields were assigned to the ligand and protein, respectively. The RESP charges (Bayly *et al.*, 1993 ▶) were assigned to each ligand. Charged states were assumed for nicotinic acid and benzamidine molecules. The geometry of each complex structure was optimized by *Amber* (Pearlman *et al.*, 1995 ▶) with 500 steps of the steepest descent energy minimization constraining the protein position with 100 kcal mol^−1^ Å^−1^. The optimized structures were then subjected to the single-point binding energy calculation by the MM-PBSA method. Dielectric constants of 4 and 80 were assigned to the solute and solvent, respectively. No distance cut-off for non-bonded interactions was applied. The binding energy Δ*G*_bind_ was finally obtained by adding the ligand deformation energy to the MM-PBSA value.

## 3. Results and discussion

### 3.1. Overall structure of CK2α

With a resolution of 1.06 Å, the human CK2α structure is the best resolved CK2α and protein kinase structure published to date. The CK2α structure had heretofore been previously determined at 1.3 Å resolution (Ferguson *et al.*, 2011 ▶), and the ephrin receptor kinase A3 structure (EphA3) at 1.07 Å (Davis *et al.*, 2008 ▶). The electron density map at a high resolution of 1.06 Å showed that most amino acid residues of CK2α were well ordered at the atomic level and the only partially dis­ordered section of the backbone structure was the tip of the glycine-rich loop around Tyr50. This loop has no unanimous conformation in the reported structures.

**Figure 1:**
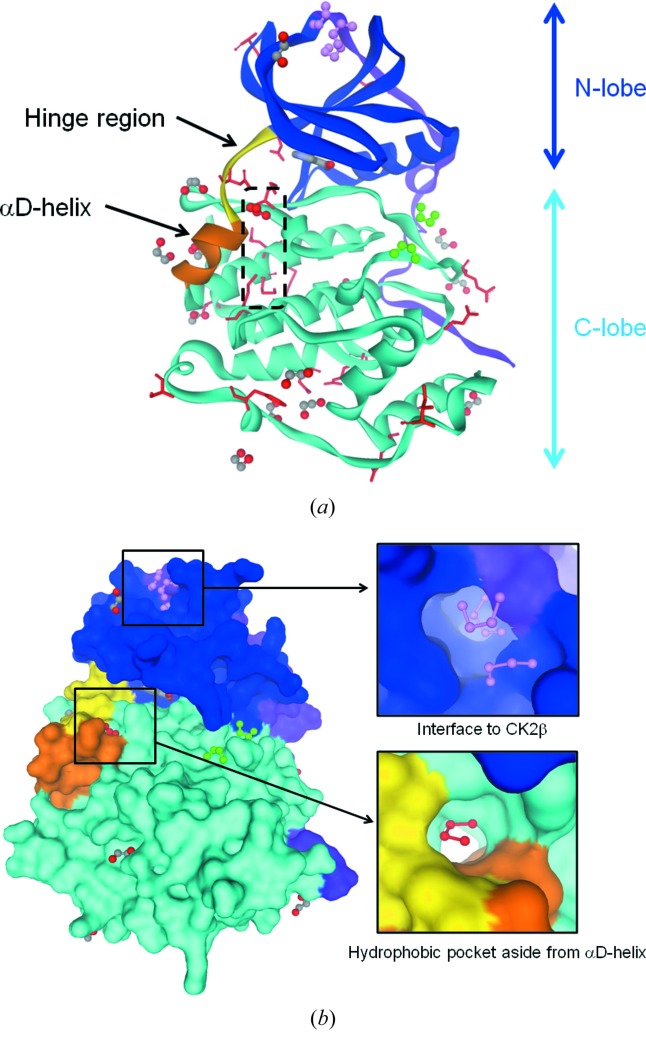
Overall structure of CK2α at 1.06 Å resolution. (*a*) A ribbon model. The five alternative conformations were observed in the interior of the molecule (black dashed box). (*b*) A solvent accessible surface. The N-terminal extension, N-lobe, hinge region, αD-helix and C-lobe are displayed by violet, blue, yellow, orange, light-blue ribbons, respectively. The nicotinic acid and ethylene glycol molecules are shown as ball and stick models. The alternative conformation loci are indicated by the red side chains.

The crystal structure of CK2α has a typical kinase fold consisting of N- and C-lobes, which are connected by a hinge region (Glu114–Asn118) (Fig. 1*a* ▶). The N-terminal extension of CK2α was found to be tightly bound to the two lobes through an aromatic cluster and a number of hydrogen bonds. At the interface of the N-terminal extension to the activation loop (Asp175–Ser194) in the C-lobe, an aromatic cluster is formed between Tyr23, Trp24 and Tyr26 on one side, and Phe181 and His183 on the other. The backbone nitrogen of Ala10 forms a hydrogen bond with the hydroxyl group of Tyr182 and the amino group of the Asn16 side-chain forms hydrogen bonds with the backbone oxygen atoms of Gly151 and Tyr182. In addition, the activation segment was observed to interact with the αC-helix *via* three hydrogen bonds. The amino group of Lys77 forms a hydrogen bond with the backbone oxygen of Gly177, and the N^η1^ atom of Arg80 forms hydrogen bonds with the backbone oxygen of Ala179 and the carboxyl group of Asp180, which is also interacting with the N-terminal extension residue Tyr26. Thus, the activation segment and the αC-helix were clamped in the active conformation because of close contacts to the N-terminal extension.

**Figure 2:**
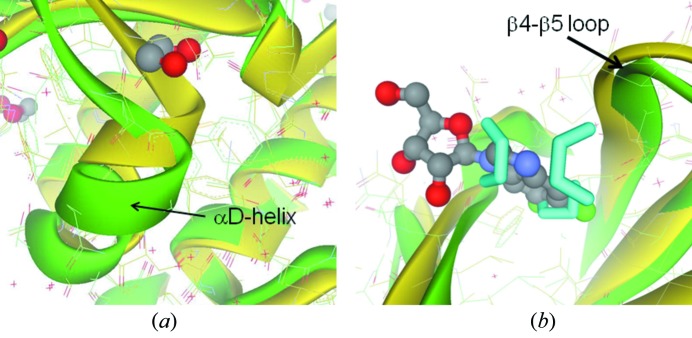
Superimposition of the 1.06 Å resolution structure (green) onto the DRB–CK2α complex structure (yellow). (*a*) The αD-helix region. (*b*) The interface region of CK2α with CK2β.

The αD-helix region (Asp120–Thr127), which is known to display various conformations, adopts the open form in this structure instead of the closed form observed in the DRB complex (Fig. 2*a* ▶) (Raaf *et al.*, 2008*b* ▶). Consequently, the deep hydrophobic pocket was revealed by the αD-helix moving (Fig. 1*b* ▶). On the other hand, the β4–β5 loop involved in the interaction with the CK2β subunit forms the closed hydrophobic pocket similar to that seen in the DRB complex (Raaf *et al.*, 2008*b* ▶) (Fig. 2*b* ▶). The C-terminal carboxyl group plugs in the cationic P+3 recognition site, which recognizes the aspartic acid located at the third residue from the phosphorylation residue (P+0). The electrostatic interaction likely allows the C-terminal strand to be well structured and extrude from the main body.

Furthermore, the high-resolution residual electron density map clearly depicted the 21 loci of alternative conformations (Fig. 1*a* ▶), whereas only four alternations had been defined previously in the 1.3 Å resolution structure (Ferguson *et al.*, 2011 ▶). Among the 21 loci, the hydrophilic side-chains of 13 residues, *i.e.* Glu32, Asn62, Glu114, Glu139, Glu187, Asp205, Glu252, Asp256, Ser287, Asp302, Arg306, Ser311 and Thr326, extrude from the protein surface and the three hydrophobic residues, Ile272, Phe284 and Met319, are spatially arranged on the respective hydrophobic protein surface. On the other hand, a total of five alternations are located in the interior of the molecule (Fig. 1*a* ▶). Three alternations at Asn118, Met163 and an ethylene glycol molecule congregate around the hydrophobic deep pocket lateral to the αD-helix. The alternations at Met137 and Met225 render a degree of plasticity to the hydrophobic cluster corresponding to an underlayment of the αD-helix involving Ile133, Ile140, Met221, Leu222 and Ile226. These interior structural alternations underpin the biological-requisite flexibility of the αD-helix, as previously reported (Yde *et al.*, 2005 ▶). Moreover, the final structure included a niacin molecule, 19 ethylene glycol molecules and 346 water molecules, which are illustrated in detail in the following sections.

### 3.2. ATP binding site

**Figure 3:**
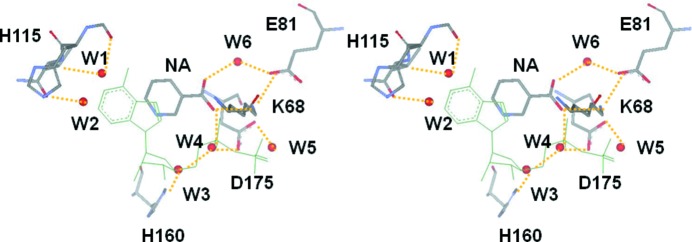
A stereo view of the ATP binding site. The nicotinic acid (NA) and six water (W1–W6) molecules bind to the ATP binding site *via* hydrogen bonds (orange dotted lines) instead of ATP. The ADP molecule in the superimposed 1.3 Å resolution structure (PDB ID 3nsz) is indicated by green lines as a reference.

The six water molecules (W1–W6) bind to the ATP binding site, involving the adenine, ribose and phosphate sub-sites, *via* hydrogen bonding (Fig. 3 ▶). The water molecules W1 and W2 are located at the adenine sub-site and hydrogen-bond with the backbone of Glu114 and Val116 in the hinge region, respectively. Another water molecule, W3, binds at the ribose sub-site by forming a hydrogen bond with His160. Two other water molecules, W4 and W5, reside in the phosphate sub-site and form hydrogen bonds with Asp175 and the niacin molecule. Finally, a well ordered water molecule, W6, displaying a low *B*-value of 9.04 Å^2^ compared with the all-atom mean *B*-value of 14.35 Å^2^, is located in the bottom of the ATP binding site and forms part of the circular hydrogen network consisting of Lys68, Glu81 and niacin (Fig. 3 ▶).

**Figure 4:**
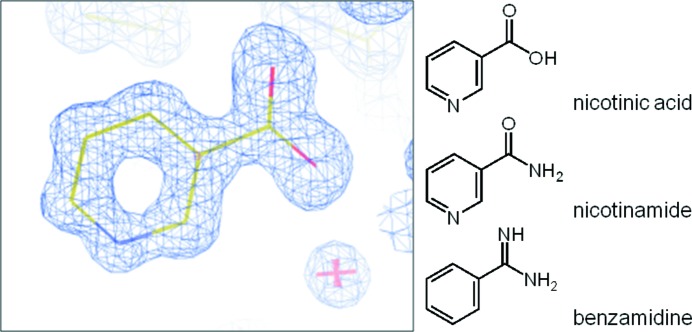
Electron density corresponding to a niacin (general name for nicotinic acid and nicotinamide) molecule in the ATP binding site.

**Figure 5:**
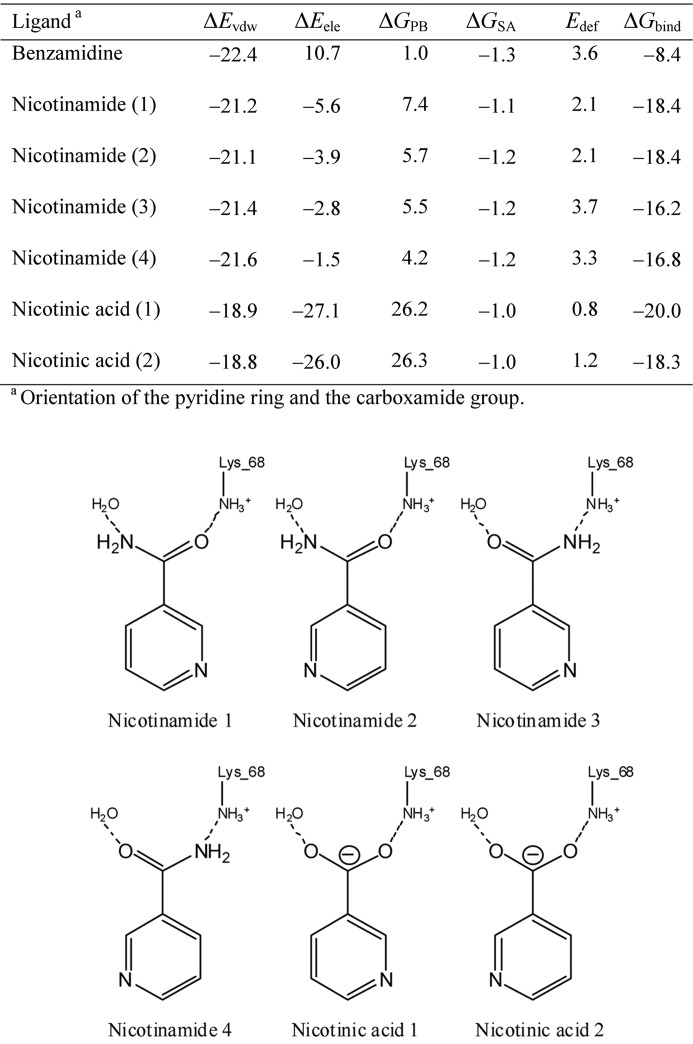
Binding energy, Δ*G*_bind_, and its components of the assumed ligand with CK2α (all in kcal mol^−1^).

Despite the absence of ATP during the crystallization process, an equivalent residual density occurred that we interpreted as niacin (Fig. 4 ▶), a general name for nicotinic acid and nicotinamide, which are general components present in the culture medium, although previously the density had been interpreted as benzamidine, a component of the inhibitor cocktail used in their purification procedure (Yde *et al.*, 2005 ▶). The probability that it was benzamidine was excluded, because the residual density was present without use of the inhibitor cocktail in our purification procedure, and by the computational investigation revealing that the binding affinity of benzamidine with CK2α was significantly small when compared with niacin (Fig. 5 ▶). Calculated binding energies also suggest that nicotinic acid predominantly binds to the ATP site rather than nicotinamide in our experimental environment, although nicotinamide is sufficient for moderate binding (Fig. 5 ▶). The crystal structure shows that the pyridine moiety of the nicotinic acid molecule is surrounded by several hydrophobic residues. These residues include the two gatekeepers, Phe113 and Ile174, while the residues Val53 and Val66 extrude from the N-lobe, and the carboxylic moiety of the ligand forms a salt bridge with Lys68 and two water-mediate hydrogen bonds with Glu81 and Asp175 (Fig. 3 ▶) with the apparent similarity to the other inhibitors possessing this moiety (Kinoshita *et al.*, 2011 ▶; Hou *et al.*, 2012 ▶). On the other hand, the binding model revealed that the carboxamide group of nicotinamide, putative secondary binder (Fig. 5 ▶), consistently formed a hydrogen-bond network identical to the carboxylic acid group of nicotinic acid.

### 3.3. Ethylene glycol molecules probe the CK2α protein surface

Ethylene glycol was used as a crystallization precipitant, and served as a chemical probe of the hydrophobic and hydrophilic protein surface. The high-resolution residual electron density map revealed that 19 ethylene glycol molecules bound on the protein surface. Among them, eight molecules merely occupy the gap in the crystal packing and have no significant interaction with the protein. On the other hand, another ethylene glycol molecule likely stabilizes the partial conformation of the αD-helix consisting of Asp120–Thr127 through mediating the hydrogen-bond relay between Thr119 and Glu167. The other five molecules individually cover the locally exposed hydrophobic areas and tentatively turn them to be hydrophilic. Among them, a disordered ethylene glycol molecule is located at the top of the putatively druggable hydrophobic pocket because it is lateral to the αD-helix forming rim of the ATP binding site [Figs. 1(*b*) ▶ and 2(*a*) ▶]. Therefore this pocket is useful for introducing a hydrophobic functional group to inhibitors. The rest of the five ethylene glycol molecules occupy physiologically significant sites: the CK2β binding interface and the substrate recognition site. Three ethylene glycol molecules bind to the CK2β interface (Fig. 1*b* ▶), in which the DRB or glycerol molecule had been observed previously (Raaf *et al.*, 2008*b* ▶). The superimposition of these compounds likely provides a structural basis to construct molecules that modulate with the CK2α/CK2β interaction (Fig. 2*b* ▶). Furthermore, two ethylene glycol molecules bind to the putative P+1 and P+3 recognition sites *via* hydrogen bonds with Lys198 and Arg80, respectively, in which a sulfate or chloride anion had been observed in previous crystal structures (Raaf *et al.*, 2008*b* ▶; Yde *et al.*, 2005 ▶).

## 4. Conclusions

We have determined the crystal structure of human CK2α to the highest resolution (1.06 Å) observed for a kinase structure to date. The high-quality electron density map explicitly conferred the individual atom positions in a large portion of the protein and thus the undefined or misassigned loci in previous investigations were resolved as alternative conformations. Although the αD-helix possesses various definite or indefinite conformations in the same crystal system (unpublished data), this crystal structure revealed that the αD-helix is distinctly structured. Moreover, this helix adopts the open form with an ensemble of interior alternative conformers (Fig. 1 ▶), which is a presage for the flexible mechanism in the αD-helix responsible for the dual usage of GTP and ATP by the kinase. The experimental artifacts, involving niacin and ethylene glycol molecules as well as water molecules, were found at the protein surface and confer striking hints for rational drug discovery. The niacin, which remained bound to the active site during the purification procedure, is available as a useful druggable fragment. It is a remarkable finding from computational results that the carboxamide group of the nicotinamide is probably allowed to locate in the equivalent position to the carboxyl group, which had been identified in previous studies (Kinoshita *et al.*, 2011 ▶; Hou *et al.*, 2012 ▶). The ethylene glycol molecules served as chemical probes for the CK2α protein surface, similar to the buffering reagent or organic solvent approach seen for elastase (Kinoshita *et al.*, 2006 ▶; Mattos *et al.*, 2006 ▶). Actually, several ethylene glycol molecules bound to biologically significant positions involving the CK2α/CK2β interface, substrate recognition sites and the remarkable druggable pocket lateral to the αD-helix. Together with the ligated CK2α structures previously reported, the results derived from the binding of ethylene glycol probably promote structure-based and/or fragment-based drug discovery, thereby providing a powerful complementary strategy to guide computational methods currently in development for binding site determination, ligand docking and design. 

## Supplementary Material

PDB reference: 3war

## Figures and Tables

**Table 1:** Data collection and refinement statistics

Data collection	
Space group	*P*2_1_2_1_2_1_
Unit cell (Å)	*a* = 51.45, *b* = 78.31, *c* = 80.14
Observed reflections	869858
Unique reflections	137982
Resolutions (Å)	56.00–1.06 (1.08–1.06)
Completeness	94.1 (74.1)
*R*_merge_ (%)†	8.1 (57.3)
*I*/σ(*I*)	28.2 (1.4)

Refinement statistics	
Resolution (Å)	56.00–1.06
Reflections	131177
Total atoms	3358
*R*_work_ (%)‡	14.0
*R*_free_ (%)§	16.5
R.m.s. deviations	
Bond length (Å)	0.030
Bond angle (°)	2.2

Values in parentheses are for the highest-resolution shell.

† *R*_merge_ = Σ_*h*_Σ_*j*_|*I*_*hj*_− 〈*I*_*h*_〉|/Σ_*h*_Σ_*j*_|*I*_*hj*_|, where *h* represents a unique reflection and *j* represents symmetry-equivalent indices. *I* is the observed intensity and 〈*I*〉 is the mean value of *I*.

‡ *R*_work_ = Σ|*F*_obs_ − *F*_calc_|/Σ*F*_obs_, where *F*_obs_ and *F*_calc_ are the observed and calculated structure-factor amplitudes, respectively.

§ The *R*_free_ value was calculated with a random 5% subset of all reflections excluded from the refinement.
